# Miscellaneous Facts and Remarks

**Published:** 1799-05

**Authors:** 


### *Miſcellaneous Facts and Remarks.*

Much has been ſaid and written, within the laſt fifty years, on the internal uſe of *phoſphorus*, and its medicinal properties. We think it needleſs to waſte much room and time on a ſubſtance, which has been as frequently recommended as it has been forgotten, and placed on the condemned liſt. To ſatisfy our readers, however, reſpecting the nature and effects of this powerful remedy, we ſhall briefly extract the opinion of an eſteemed modern writer, Dr. GESENIUS, from his excellent "*Manual of Therapeutics*," in German, 8vo. 1796. "This ſubſtance," ſays he, "phyſicians have again lately ventured to preſcribe; they have praiſed its ſucceſs in different caſes, and again remained ſilent reſpecting its further uſe. According to the obſervations of reſpectable practitioners, it cannot be denied, that phoſphorus poſſeſſes a peculiar power of exciting and animating the nervous ſyſtem. It has been aſſerted, that, in doſes of two, three, and more grains, it has been productive of ſalutary effects in the moſt critical caſes of putrid typhus fever, nervous apoplexy, gout, and epilepſy. As it cannot be adminiſtered in ſubſtance, on account of its burning quality, it has been diſſolved in oil; and, probably with a view to increaſe its ſtrength, ſome of the water in which it was preſerved, has been added to the ſolution. In my opinion, it is a medicine we may diſpenſe with, as we poſſeſs other *more ſafe* nervines. I feel no inclination ever to employ this remedy, notwithſtanding the regard I profeſs for for thofe who recommend it; and any perfon who is ftill anxious to try the effects of phofphorus, I would advife previoufly to confider what Weikardt, from his own experience, has ingenuoufly communicated to us, refpecting the ufe of it, in his "Mifcellaneous Medical Writings," in German, Number IV. p. 105, *et feq.* The beft writers on this fubject are, "A. Vater, *refpond. Mentz*, Differtatio de Phofphori medicamenti loco affumpti virtute medicâ." *Vitemb.* 1751.—and "T. Josephi Differtatio de Phofphori ufu interno." 8vo. *Helmft.* 1789.

---

*Naufea* and *vomiting* are frequently diftreffing fymptoms during pregnancy. Any remedy, therefore, calculated to relieve, or at leaft mitigate, fuch complaints, muft prove highly acceptable both to the general and profeffional reader. Dr. Marcard, phyfician to the Court of Oldenburg, and author of a claffical work, "*On the practical ufe of baths*," publifhed about four years ago, in the German language, advifes pregnant women to drink fimply the *acidulated mineral waters*, particularly that of *Seltzer*. Dr. M. accompanies this advice with the following remark: "Although it produce only a palliative cure, yet, as the remedy can be as often repeated as there is a neceffity for it, and as thefe fymptoms are limited to females in a certain ftate, the relief thus afforded is of equal benefit as if it performed a radical cure." We fhall only add, that a *radical cure*, in this inftance, is not within the power of art, as the caufe of the complaint cannot, or ought not, to be prematurely removed.

---

Profeffor Hufeland, of Jena, ftrongly recommends the ufe of *narcotic remedies* in *chronic inflammation of the eyes*, efpecially in children. It is of the greateft importance, fays he, in his valuable work, '*on infantile difeafes*,' to pay particular attention to the increafed and preternatural irritability of the difeafed part, in the chronic and particularly in fcrophulous opthalmia of children; for this irritability alone may, even after the material caufe is removed, occafion confiderable pain, fpafmodic ftricture, and inflammation. Nay, the caufe itfelf frequently cannot be removed, until the fpafm be relieved by fuch anti-fpafmodic remedies which, in a manner, blunt and reduce the fenfe of feeling. Even mercurial remedies, when exhibited in fuch cafes, with a view to fupprefs the caufe of the malady, frequently operate as new ftimuli, unlefs they be rendered milder, more congenial to the conftitution, and confequently more falutary, by combining them with narcotics."—The Profeffor relates a cafe of violent humoral opthalmia in a child, whofe eyes were fo completely clofed, that they could not be opened by external force. He directed frequent poultices, confifting of the leaves of of hyoscyamus boiled in milk, to be applied to the eyes, and repeated mercurial laxatives to be taken internally. The patient discharged a number of worms, and the inflammation of the eyes entirely subsided. The eyes opened spontaneously, and, excepting a slight dimness, completely recovered.

---

The same author describes an extraordinary case of cancer on the lip of a scrophulous boy, eleven years of age, who, when two years old, bit his under lip in a fall; so that a small nodule appeared at first, which in process of time degenerated into a *confirmed cancer*. With this child all the quacks and old women of the neighbourhood had tried their schemes, when he was submitted to the medical treatment of Hufeland. After having prescribed the belladonna, rhubarb, camphor, cicuta, antimony, and mercurial preparations, with little or no effect, the professor at length directed him to take fifteen drops of the *volatile spirit of sal ammoniac*, much diluted with water, three times a day, and to increase the doses with four additional drops every day. At the same time, he ordered him to drink a decoction of the young sprigs of the pine-tree, (turion. pini—an excellent antiscorbutic and antiscrophulous medicine,) together with a tea-cupful of the freshly expressed juice of carrots, every morning, and to make use of a luke-warm saponated bath, every other day. Besides these, a solution of one scruple of antim. tartar. in four ounces of water, was prescribed, of which the patient, frequently through the day, took a little at a time in his mouth, and moistened with it the affected part of the lip. "This solution," says Mr. H. "externally used, is an active resolvent and stimulant remedy, from the immediate absorption of which, by the lymphatic vessels, I promised myself great benefit."

Scarcely had these remedies been regularly continued for four weeks, when an obviously favourable change took place in the disorder; the scirrhosity became softer and smaller; the scabs began gradually to diminish, and the diseased parts became more even and smooth. The dose of the ammoniacal drops was increased to forty, taken three times a day, without the least inconvenience; and after having duly persisted in the use of the remedies for *three months*, the cancerous lip was reduced to its natural state, and all other callosities about the glands of the neck, had completely disappeared.

---

Professor Thunberg, the celebrated Swedish traveller, has enriched the materia medica with a remedy which deserves the attention of practitioners, in a more than ordinary degree, and which has been found to possess pre-eminent virtues as an anodyne, antispasmodic, and stimulant. We allude to the *oil of cajeput*, a substance which, although introduced into several

ſeveral modern pharmacopoeias, is ſo little employed in actual practice, that it is ſcarcely known by name to moſt medical men, and is ſtill more rarely kept in the ſhops, even in the metropolis.

According to the obſervations of Thunberg, who is a man of unqueſtionable authority, this oil has been ſucceſsfully preſcribed in chronic *inflammations of the eyes*. He directs a few drops of this volatile ſubſtance to be poured on a ſoft white linen cloth, which are allowed firſt to evaporate, while held pretty cloſe to the eyes, and afterwards the ſame cloth is to be tied over theſe organs during the night.

In the *gout*, as well as in *acute rheumatiſm*, it has proved of immediate ſervice, by anointing the affected part with this ethereal oil, which has a remarkable tendency to open the pores, and diſſipate, in the words of the profeſſor, "the arthritic and rheumatic matter."

In violent *head-achs* it has ſometimes afforded ſpeedy relief, by rubbing the temples with it, and inhaling it through the noſtrils.

In caſes of *tooth-ach*, from whatever cauſe, ſays Thunberg, the affection may proceed, whether from a carious hollow tooth, or rheumatic acrimony, catarrh, &c. the cajeput oil has generally been efficacious in removing the complaint, if dropped on lint, and placed in the cavity of the tooth, upon the tooth itſelf, or even at the ſide of it. Although theſe accounts may appear ſomewhat exaggerated, yet, in ſeveral caſes of odontalgia, and acute rheumatiſm, we are from experience convinced of its happy effects.

---

From a diſſertation on the *ſick head-ach*, read before the Medical Society of Hartford county, Connecticut, by Dr. Nathaniel Dwight, we extract the following particulars:—After noticing the general ſilence of medical writers on this ſubject, and remarking that Dr. Fothergill alone has formally conſidered it; that the ſick head-ach is a diſeaſe of uncommon ſeverity, and generally ſuppoſed incurable; that therefore a remedy adequate to its radical cure muſt deſerve to be highly valued; and that the efficacy of the one which he ſhall propoſe can be verified by many perſons, and in particular by Drs. Fiſh and Cogſwell, of Hartford, America; the author gives an accurate pathological deſcription of this malady, which however our limits do not admit to be inſerted, ſo that we muſt immediately proceed to the practical part of his treatiſe.

In diſcourſing of the method of cure, Dr. Dwight maintains, that "*vegetable acids* in a ſupereminent degree, correct the *(morbid)* acid of the ſtomach." Mineral acids, he admits, poſſeſs a ſhare of this property, but in an inferior degree.

The

The next object of inquiry is, what vegetable acid is preferable to all others? and in what form?—In anſwer to this, Dr. Dwight declares, that ample experience has eſtabliſhed the ſuperiority of the *malic acid*, or *cyder*, over every other; and he points out a remedy and mode of cure, equally remarkable for ſimplicity and efficacy. For this purpoſe, cyder muſt be well gone through the firſt fermentative proceſs, and no farther. It ought to be free from all taſte of the caſk, and all other impurities. A quantity of it, from half a gill to half a pint, ſhould be drank on an empty ſtomach, in the morning, from five to fifteen minutes before breakfaſt.

The efficacy of cyder, in the cure of the ſick head-ach, leads Dr. Dwight to the mention of its ſucceſs in the cure, or rather prevention of another diſeaſe, which he ſuppoſes to depend on the ſame cauſe, the *bilious colic*. That the ſick head-ach, and this ſpecies of colic, depend on the ſame cauſe, differently applied, he infers, 1ſt. from their affecting perſons of the ſame temperament, the *bilious*; 2d, from their both obſerving ſimilar periodical returns; 3d, from the circumſtance of perſons who, in the courſe of their lives, have been ſubject to both complaints for years, having uniformly experienced an exemption from one, during the prevalence of the other, alternately; and 4th, from the efficacy of cyder in the cure of colic, as well as head-ach. "The evidence of its efficacy," continues Dr. Dwight, is this: The cyder has been drunk by perſons for months together, with entire relief from the colic, who, before they began with the remedy, were ſubject to paroxyſms of it every few weeks. After ſeveral months had expired, the cyder was laid aſide, and the colic returned with as great frequency and ſeverity as before. The remedy was reſumed, and the colic again diſappeared."

---

Dr. Edward Miller, of New-York, has lately publiſhed an elaborate "*Inquiry concerning cutaneous Perſpiration, and the Operation and Uſes of ſudorific Remedies*", which we find inſerted in the Medical Repoſitory, Vol. II. No. I.—As this eſſay extends to a conſiderable length, we can at preſent only extract from it a few paſſages of a *practical* tendency, which we conſider ſufficiently intereſting to engage the attention of the reader. After having ably commented on the great importance of the function he treats of, as well as the relation which the cutaneous perſpiration bears to the critical ſolution of febrile diſeaſes, Dr. Miller gives the following opinion on this ſubject, as the reſult of his inquiry:

"That ſudorifics cannot be uſefully employed as a general remedy in fevers, is apparent from the fatal courſe purſued by many of theſe diſeaſes, notwithſtanding the moſt copious, univerſal, and continued ſweats, ſpontaneouſly neouſly taking place. The memorable ſweating ſickneſs, which firſt appeared in England, towards the cloſe of the fifteenth century, and was one of the moſt fatal epidemics on medical record, affords ample proof of this poſition.

"On the whole it may be concluded, that much of the uſe of ſudorifics has ariſen from miſtaken doctrines, concerning the nature of perſpiration and of fever, particularly from the erroneous opinions, that the matter of perſpiration is excrementitious; that its occaſional obſtruction is noxious; that it ought, as much as poſſible, to be eliminated from the ſyſtem; and that it is only carried off, in conſiderable quantity, when diſcoverable by ſight or touch.

"It may be alſo concluded, that ſudorific remedies, eſpecially thoſe of the more powerful kind, are, in general, highly unſafe, and calculated to augment the violence of inflammatory and malignant fevers; and, that though they may ſucceed in ſome caſes of leſs violence, or by a favourable concurrence of circumſtances, yet they are ſo conſtantly liable to produce miſchief, and exaſperate the diſeaſe, that the abuſe, on the whole, muſt be pronounced greatly to overbalance the uſe."

---

Dr. DEWEES, Lecturer of Midwifery in Philadelphia, has in the ſame periodical work, publiſhed ſome '*Obſervations on the uſe of the warm bath, in caſes of laborious parturition;*' in a letter addreſſed to Dr. E. H. SMITH, of New York, lately deceaſed; from which the following paſſage we deemed worthy of being extracted:—

"The warm bath is by no means a new remedy in that ſpecies of labour, in which I have thought blood-letting of ſo much conſequence. Few writers on the ſubject of midwifery have failed to mention it among other means to overcome the rigidity of the ſoft parts in laborious labours, that does not depend on a malconformation of the pelvis. The French accoucheurs, more particularly, make frequent mention of it; yet none of them, that I am acquainted with, have laid any particular ſtreſs upon its virtues in theſe caſes, or place any great dependence on its effects. It has been rather recommended as a probable, than as a certain remedy, and ſtands upon much the ſame footing as opium—ſometimes perhaps ſucceeding, but much more frequently failing. With me it has ever been of little or no conſequence; nor can I obtain a more favourable character of it from the friends I have conſulted. The reſult then, of my experience, inquiries, and obſervation, may be reduced to three heads: 1. its being almoſt always inconvenient; 2. its being ſometimes ineligible; and, 3. its always being limited and uncertain in its effects."

Cit.

Cit. Louis Valentin, of Montpelier, has communicated to the editors of the '*Medical Repoſitory*,' a concluſive anſwer to the queſtion:—"*Whether any, and what, effect is to be attributed to the greater or leſs quantity of variolous matter introduced into the ſyſtem by inoculation? &c.*" The ſubſtance of this anſwer we give in his own words:—

"I never perceived any difference, with reſpect to the intenſeneſs of the fever, and the number of puſtules, when I made many punctures, and introduced the greateſt quantity of freſh matter I poſſibly could. Whether all the limbs, or ſeveral parts of them, be inoculated, at the ſame time, with the thin matter which firſt appears in the puſtules, and has a more certain effect than that which is maturated, or whether only one puncture be performed, is a matter of indifference. I never make inciſions: the inconvenience and trouble attending them being ſufficiently known. I make punctures, and never draw blood.

"I do not think that a milder diſeaſe is communicated by diluting the variolous matter, which is not too old; for here is my dilemma: either the matter which is to be inſerted has the requiſite qualities or not. If it be liquid, juſt gathered, and immediately inſerted—if the ſubject have all the neceſſary diſpoſitions for the abſorption and unfolding of the diſeaſe, one atom of virus only is as powerful as many drops, for the purpoſe of inoculation. If the dilution of dry and new matter be not in reaſonable proportion, or if it be rendered too liquid with water, it may have no effect; likewiſe, when undiluted, it is incapable of being abſorbed; when too old, of acting, and producing the deſired effect. But, in all caſes, ſhould the ſmalleſt particle imaginable be abſorbed, it will be ſufficient to put in motion what we call the diſpoſition to that diſeaſe, as much as when a more conſiderable maſs of matter is inſerted. This may be eaſily conceived and illuſtrated by the following compariſon: does not every one know, that one grain of powder ſet on fire in the pan of a gun, produces the exploſion of the whole charge, as well as when the pan is filled with powder? Surely, the exploſion in both caſes is neither more nor leſs conſiderable; therefore, the quantity of inſerted matter has no influence in producing the number of puſtules."

---

We cannot better conclude this department of our Journal, than by giving the reader a conciſe view of a ſubject, which has lately excited conſiderable attention among the medical practitioners of this country. It will be readily conjectured that we allude to the uſe of the *digitalis* in the cure of conſumption.

Dr. N. Drake, of Hadleigh, Suffolk, in a letter to Dr. Beddoes, dated February 21ſt, 1799, inſerted at full length in a late publication, intitled, '*Contributions to Medical and Phyſical Knowledge, collected by Thomas Beddoes, M. D.*' has furniſhed us with ſeveral valuable '*Obſervations on the uſe of the Digitalis in Pulmonary Conſumption, with two caſes in which it proved permanently ſucceſsful.*' After having prefixed a ſhort medical hiſtory of the digitalis, Dr. Drake proceeds to ſtate his views in preſcribing this plant, in caſes apparently deſperate.

As our limits do not admit of long extracts, we ſhall only obſerve, at preſent, that, although we would not implicitly ſubſcribe to the reaſonings on which the indications of cure ſeem to be eſtabliſhed, namely, "*that pus, when expoſed to atmoſpheric air, rapidly attracts oxygen; that an acid of a peculiar kind is generated; that hectic fever, the effect of the abſorption of aërated matter, is produced; that as an ulcer of the lungs is perpetually expoſed to a ſtream of air, and of courſe an ichorous poiſon continually forming by the union of oxygen with ſecreted matter, an important curative proceſs would ſeem to ariſe, from promoting abſorption ſo rapidly from the ſurface of the diſeaſed parts, that the pus ſhall be taken up as ſoon as ſecreted, and conſequently its combination with oxygen prevented:*" yet we cannot but with pleaſure and ſatisfaction congratulate Dr. Drake, and the medical world at large, on the happy, and, we fervently wiſh, *permanent* ſucceſs, of this old, almoſt abandoned remedy, in conquering the moſt formidable, and hitherto ſuppoſed *invincible* diſorder.

The preparation of the digitalis beſt adapted to that purpoſe, appeared to be the *ſaturated tincture*, of which Dr. Drake ordered to be taken, at the commencement of its uſe, in the firſt caſe, only fifteen drops, twice a day; and in the ſecond, twenty: in the former, the doſe was gradually increaſed to one hundred drops; in the latter to ninety-ſix,—" with ſafety; and in patients very debilitated, before either ſickneſs or irregularity of the circulation appeared; and even then, theſe ſymptoms proved of little moment, as the firſt was ſpeedily removed, and the ſecond produced no inconvenience. During this period, all the ſymptoms of irritation and fever, cough, pain, and dyſpnœa daily grew better, and at length altogether retired. On the quantity and quality of expectorated matter, the digitalis ſoon exerted a moſt remarkable effect, either promoting its abſorption, or dimiſhing its ſecretion, or perhaps both, in a rapid manner, whilſt at the ſame time it deprived it of its fœtor.

" What, however, I conſider as of moſt importance in theſe caſes, and to which perhaps we are alone indebted for a cure, is the demonſtration of the poſſibility of *retarding the circulation for weeks together,* by the uſe of this this medicine." p. 484. " I may, I think, without heſitation affirm, that an *early* exhibition of the ſaturated tincture in conſumptions, will, *in general*, prove ſuccefsful; and even when the diſeaſe is far advanced, provided the patient has but ſtrength ſufficient left to endure a gradual depreſſion of the circulation, a reſult equally fortunate may be expected. That this can be done, even in circumſtances of debility, to an extent adequate to effect a cure, and without either ſickneſs, languor, or loſs of appetite, the caſes now appended will ſatisfactorily atteſt." p. 487.

---

Equally intereſting, and perhaps better founded *in theory*, is the account given by Dr. R. FOWLER, in the ſame '*Collection*,' and on the ſame ſubject. To juſtify our aſſertion, in the latter reſpect, we ſhall cite Dr. Fowler's own words:—

" As I had" ſays this intelligent phyſician, " frequently ſeen large doſes of the digitalis given by others, and had, myſelf, ſtill more frequently given it in dropſical caſes, without ever obſerving any of thoſe uncontrolable and dangerous effects which are ſaid to deter many from its uſe, my mind was perfectly at eaſe as to its probable effects in phthiſis, and the more ſo, as its power of repreſſing arterial action, and inducing debility, from which we have moſt to apprehend in dropſy, was the very quality from which, properly directed, I hoped to derive moſt advantage here.

" My attention was, indeed, firſt directed to it, as a remedy likely to be uſeful in phthiſis, by its almoſt uniform effect of rendering the action of the arteries more ſlow than natural, at the ſame time that it appears to excite that of the abſorbents. It has long been known, that diſeaſed parts of the body may be removed by depriving them of *all* ſupply of food from the arteries; and it is now known, that where this cannot with ſafety be attempted to ſo full an extent, on account of the intimate connection ſubſiſting between the part to be removed, and ſuch as we wiſh ſhould remain, that the ſame effect may be produced by diminiſhing, to a certain degree, the arterial ſupply of the part, at the ſame time that we leave the action of the abſorbents in full force. This is the purpoſe ſo ably effected by Mr. HUNTER's ſcientific operation for the cure of popliteal aneuriſm: and I confeſs that I was not, and that I ſtill am not, without hope, that ſomething analogous to this may be effected by the operation of digitalis on tubercles in the ſubſtance of the lungs. But my expectations of ſucceſs had a better foundation than reaſoning *a priori*".

From a review of the different caſes ſtated by Dr. Fowler, we find that *four* of his *out*-patients completely recovered from the uſe of digitalis, and

*five*

*five* were in a fair way of recovery, when his account was transmitted to the press. Of the second class, namely, the *in-patients* of Dr. Fowler, together with those he attended in private practice, there are not less than *seven* who obtained permanent relief, and *two* whose cases were of a doubtful issue, and *one* only who "was a very intemperate man, and died a victim to his own imprudence".—The formula prescribed by Dr. Fowler, is the following: Folior. digitalis purpureæ recentium unc. ii. coque ex aq. puræ, lb. i. ad colaturæ unc. vii. ss. et adde tinct. cardamom. unc. ss. Of this decoction Dr. Fowler generally directed his patients to take half an ounce, twice, thrice, and in a few instances four times in twenty-four hours: and although we do not discover among his patients, many such as, according to Dr. Beddoes's Nosology, might be classed under the *lost cases*, yet the success of the *new remedy* * has, upon the whole, exceeded our most sanguine hopes. It is however to be regretted, that we are not yet in possession of any cases, in which the origin and progress of pulmonary consumption is traced with *pathological accuracy;* the peculiar constitution, habit, and temperament of the individual, are duly discriminated; and all the concurrent and accidental circumstances, such as the weather, air, climate, season, temperature, &c. are distinctly recorded.

In justice to the editor of the volume from which we have derived the information imparted to us by Dr. Drake and Dr. Fowler, we shall likewise present our readers with a few extracts from the 'Addition' made to these '*Contributions*', by Dr. Beddoes himself, without any comment.

"A good

---

* It is impossible to contemplate the *rationale*, or rather the *modus operandi*, of the digitalis, as stated by the ingenious writers on that subject, without some degree of reluctance. They seem to attribute to this plant, in effect, a peculiar specific action; not as a mere emetic, nor as a stimulant, sedative, &c.; for, these effects are produced by a great number and variety of other substances equally powerful and less dangerous in their application. Indeed, it ought to be remembered, that the late celebrated Professor STOLL, of *Vienna*, in his '*Ratio medendi*', has furnished us with many instances of *incipient phthisis*, and with a few which may not improperly be termed '*lost cases*', where he almost uniformly prescribed the smallest doses of antim. tartaris., or the sulphur antim. præcipit., so as to produce continued slight nausea, with singular and unequivocal success. As long, therefore, as it remains to be determined, that the digitalis *alone*, and not conjointly with other medicines, has uniformly cured that formidable disorder, pulmonary consumption, in its more advanced stages; and lastly, that this plant produces effects on the human system different from those of *all other emetics*,—we cannot but suspend our opinion respecting its extraordinary virtues. In the mean time, we beg leave to disavow our belief in *any specifics whatever*; being firmly persuaded, that there exists no substance in nature, which can be considered homogeneous, or even analogous, in a physiological sense, to the elementary constituents of the human or animal body.

W.

" A good many of the cases," says he, " in which I have hitherto tried it in *effective* doses, were *lost cases*, that is, the patients were in the last stage of consumption. I have not yet been fortunate enough to rescue any patient in such a situation; but in most instances there was a great alleviation of symptoms; in none did life appear to be shortened by the medicine; in some, as far as analogy enables me to judge, it was generally protracted. P. 521.

" What is the cause of the difference between my success, and that of my two able correspondents? From Dr. Fowler's statement, it is clear, that it does not lie altogether in the *period of the disease*. I have suspected that at first, when I used the decoction, I prescribed it too largely; yet when I again refer to Dr. Fowler's reports, I am obliged to reject this supposition. In the patient whose expectoration of mucus was stopped by an attack of severe sickness, (a circumstance which inspired me with hopes of eventual success) the purulent part of the expectoration was diminished at each of the subsequent sicknesses: and the effect of sea-sickness upon many phthisical patients, forbids me to conclude, that the medicine failed from too great severity of its action. P. 533.

" My patients were all slender, delicate, puny, or feeble. They had all been *delicately educated*, in which respect they must have differed widely from *hospital patients*. Now may I not assume (what in an '*Essay on Consumption*,' I shall immediately endeavour to prove, by a copious induction of facts) that almost all the peculiarities in the mode of life, among the more opulent classes, tend to lessen the *contractile power* of the muscular fibre, and certainly not less than the rest, the contractility of the lymphatic vessels? If so, I believe it will be easily allowed, that they will be less within the power of association of motions, as well as of direct stimuli. I have long *believed it* to be a principle of the animal oeconomy, that in weak habits the ordinary or natural connection between different sets of moving fibres (or the irritative associations,) are also weak. In such cases, the stomach may be affected; the heart may be affected; yet the lymphatics of the lungs, which in less feeble or more irritable habits, are excited into action, shall continue inert. The facts which I have related, concerning the two last patients, prove that the stomach was affected in all degrees consistent with safety to life, by the digitalis; and yet, that no adequate excitement of the absorbents was produced. I do not offer this opinion for acceptance, but merely to be compared with *future facts*. When Dr. Fowler favours us with those comments, he gives reason to expect, perhaps he will help us to a better explanation. Few people are better qualified for illustrating the actions of the animal economy.

" In

" In five caſes of imminent or incipient conſumption, the uſe of digitalis has either removed the complaint, or by producing the moſt decided good effect, affords hope of ſucceſs. P. 536.

" An immediate emulation muſt be produced by theſe reports among medical obſervers. But I will beg leave to ſuggeſt to them, that the determination of the powers of digitalis, is not the only, perhaps after what has been already done, not the greateſt, object of purſuit. We may preſume, that nothing ſtands alone in nature. And a ſubſtance of ſimilar effect on the ſtomach and arterial ſyſtem, may lurk among the articles of the materia medica. It may be detected in ſome of the bitters, given in greater doſes than common. It is impoſſible here, not to think of chamomile, which, in a certain doſe, ſickens; and of horehound, which is the general domeſtic remedy in hæmoptyſis and bad coughs. Theſe, and other ſubſtances given upon the above plan, may exert virtues ſimilar to thoſe of digitalis. Chemical analyſis may furniſh light here, on which account I am glad Mr. Davy has engaged in the analyſis of the digitalis. The diſcovery of an analogous body would add prodigiouſly to future ſucceſs in the treatment of conſumption." P. 538.

---

Dr. Lentin, of Luneburg, a celebrated German phyſician, has lately publiſhed in the "*Commentaries of the Royal Society of Göttingen*", ſome remarks on the *caries oſſium*, and the cure of that diſorder. He conſiders it to proceed, on the one hand, from the chemical decompoſition of the phoſphate of lime, produced by the putrefaction of the jelly contained in the bone. In this view, he is of opinion, that the phoſphoric acid will be of advantage in this complaint, and his experience ſeems to confirm that idea. He adminiſters from ten to twenty drops of the acid internally, in a convenient vehicle; externally, one part of the ſame acid, with ſeven parts of diſtilled water. He has remarked, that the peculiar fetid ſmell of caries, by theſe means, in a ſhort time diſappears, and the cure ſpeedily ſuceeds. He adds, on the other hand, that patients affected with hæmorrhoidal ſymptoms, as well as wo men during menſtruation, were rendered ſomewhat uneaſy and irritable by the uſe of this new remedy.

---

MEDICAL

